# Effects of microencapsulated phytonutrients from fruit peel pellet on rumen fermentation efficiency, *in vitro* degradability, methane production and microbial diversity

**DOI:** 10.5713/ab.24.0660

**Published:** 2025-02-27

**Authors:** Sukruthai Sommai, Chanon Suntara, Maharach Matra, Srisan Phupaboon, Gamonmas Dagaew, Chaichana Suriyapha, Rittikeard Prachumchai, Metha Wanapat

**Affiliations:** 1 Tropical Feed Resources Research and Development Center (TROFREC), Department of Animal Science, Faculty of Agriculture, Khon Kaen University, Khon Kaen, Thailand; 2 Division of Animal Science, Department of Agricultural Technology, Faculty of Technology, Mahasarakham University, Maha Sarakham, Thailand; 3 Department of Agricultural Technology, Faculty of Science and Technology, Thammasat University, Pathum Thani, Thailand

**Keywords:** Fruit Peel, Methane Emission, Microencapsulation, Phytonutrients, Rumen Fermentation

## Abstract

**Objective:**

The current study aimed to determine the impact of supplementing fruit peel pellet microencapsulated phytonutrients (mMARABAC) on rumen fermentation efficiency, *in vitro* degradability, methane production, and microbial diversity.

**Methods:**

The experiment was completely random, and the dietary treatments were mMARABAC supplements at 0, 5, 10, and 15 mg/500 mg dry matter (DM) of substrate (rice straw and concentrate).

**Results:**

The experiment’s results demonstrated that mMARABAC supplementation significantly affected the gas production from the insoluble fraction (b), rate of gas production value (c), and potential extent of gas (|a|+b) value (p<0.05), while the soluble fraction of gas production (a) was not influenced by the treatments. Furthermore, mMARABAC increased the cumulative gas at 96 h after incubation (p<0.05) when mMARABAC was supplemented with 5 mg of total DM substrate. However, mMARABAC supplementation did not have an effect on *in vitro* degradability of dry matter (p>0.05). The supplementation of mMARABAC did not significantly affect ruminal pH and NH_3_-N (p>0.05). Additionally, there was linearly (p<0.05) decreased CH_4_ production at 24 hours of incubation. The mMARABAC supplementation to the diet did not affect the concentration of total volatile fatty acids, acetic acid (C2), propionic acid (C3), butyric acid, or the C2:C3 ratio (p>0.05). The effect of mMARABAC supplementation on *Fibrobacter succinogenes*, *Ruminococcus albus*, *Ruminococcus flavefaciens*, *Butyrivibrio fibrisolvens* and *Megasphaera elsdenii* was different between treatments (p<0.05), while the mMARABAC supplement had an effect on Methanobacterial (p>0.05).

**Conclusion:**

This study suggested that ruminants could use agricultural by-products as a source of phytonutrients by supplementing with mMARABAC at 5 mg/500 mg DM of substrate.

## INTRODUCTION

Global warming generated by greenhouse gas emissions threatens the sustainability of humans and other organisms on earth [1]. Livestock production contributes to global warming through the emission of greenhouse gases (GHG) [2]. Ruminants produce methane (CH_4_), one of the major GHGs, when microbes degrade feed in the rumen. Additional products resulting from the process of feed digestion throughout the rumen include volatile fatty acids (VFAs), carbon dioxide (CO_2_), and hydrogen (H_2_) [3]. Rumen fermentation has significant implications for animal productivity and the environment.

Phytonutrients are a large group of secondary metabolites that can be found in fruit peels and other waste products. According to Abbas et al [4], these chemicals play a crucial role in promoting animal nutrition and overall well-being. Furthermore, literature to date has shown effects of polyphenolic plant compounds on decreasing CH_4_ emission and methanogen population [5]. Methanogenic archaea in the rumen are responsible for the production of enteric CH_4_ that is released to the atmosphere by ruminant animals [6]. Thus, bioactive compound supplementation could alter ruminal fermentation and elicit changes in ruminal microorganisms, which may result in reduced enteric gas emissions. According to Wanapat et al [7], phytonutrient pellets, also known as MARABAC, originated from a variety of tropical fruit peels that contain mangosteen peel, rambutan peel, banana flower powder, and cassava starch. High levels of concentrate mixture supplementation significantly enhanced rumen pH and fermentation products., while reducing CH_4_ production. When fed at a high concentration, it can buffer rumen pH and improve rumen fermentation efficiency, particularly propionate production. MARABAC has the potential to function as an alternative dietary enhancer for the rumen and improves rumen fermentation efficiency [7]. In addition, Phupaboon et al [8] demonstrated that microencapsulation technology improves stability, enhances bioaccessibility, imparts controlled release features, improves storage and handling convenience. Encapsulation has the potential to improve production efficiency throughout the animal feed sector. Recently, Suriyapha et al [9] established that microencapsulated phytonutrients (mMARABAC) derived from lemongrass combined with dragon fruit peel pellets can enhance gas production, *in vitro* degradability of dry matter (IVDMD), and ammonia nitrogen (NH_3_-N) concentration, additionally, the microbial population and rumen fermentation improve, while the methanogen population and CH_4_ production decrease.

Therefore, the objectives of this study were to determine the effect of including increasing amounts of mMARABAC on rumen fermentation efficiency, nutrient degradability, CH_4_ production, and microbial diversity *in vitro*.

## MATERIALS AND METHODS

### Animal ethics

The experiment received official review and approval from the Institutional Animal Care and Use Committee of Khon Kaen University, in accordance with the Ethics of Animal Experimentation established by the National Research Council of Thailand (Record No. IACUC-KKU-110/66 and Reference No. 0201.2.11/73).

### Preparation of MARABAC pellet and MARABAC extraction

The product of phytonutrient pellets, MARABAC, was followed by Wanapat et al [7]. The bioactive compounds from MARABAC extract were prepared from 10 g of MARABAC pellet powder mixed with 100 mL of deionized water and extracted by using the microwave extraction method in an optimal condition of microwave power at 100 W for 10 to 15 min (until the temperature reached 60°C) according to the procedure of Phupaboon et al [8].

### Microencapsulation of MARABAC process

The microencapsulated MARABAC (mMARABAC) was formulated at a 1:1 ratio between the MARABAC extract solution and the wall material containing 2% of chitosan media dissolved in a 1% acetic acid (C2) solution as an ionic gelation technique. After being stirred for overnight, the homogeneous media was processed through the spray-drying technique (B-191 mini spray dryer; Bǚchi, Flawil, Switzerland) at optimal conditions, and the dried powders of mMARABAC were collected, hermetically sealed, and then stored at a temperature of −20°C until use in *in vitro* experiments according to the procedure used by Phupaboon et al [8], Nouri [10] and Kurek and Pratap-Singh [11].

### Estimation of total polyphenolic and total flavonoid contents and antioxidant capacity

The measurement of the total polyphenolic content (TPC), total flavonoid content (TFC), and antioxidant capability of extracts containing bioactive compounds was performed utilizing a 96-well microplate assay. The biochemical assay mode of an EnSight multimode plate reader (PerkinElmer Inc., Waltham, MA, USA) and Kaleido Data Acquisition software were utilized in this study, following the approach defined by Phupaboon et al [8]. The assay employed label-free applications. The antioxidant activity was assessed using three methods: the 2, 2-diphenyl-1-picrylhydrazyl (DPPH) radical scavenging method [12], the 2, 2′-azino-bis (3-ethylbenzthiazoline-6-sulphonic acid; ABTS) radical scavenging activity [13] and the ferric reducing antioxidant power (FRAP) method [14]. The analyses were conducted in triplicate, and the outcomes were reported as a percentage of radical scavenging inhibition and millimoles of Trolox equivalents per gram of dry matter (mmol TROE/g DM).

### Chemical composition analysis

The roughage and concentrate samples were taken, combined into composite samples, and further processed by chopping and grinding for the purpose of conducting chemical analysis. The analysis of all samples included determining DM (ID 967.03) and ash (ID 492.05) content using the standard AOAC procedures [15]. The Nitrogen Analyzer (Leco FP828; LECO Corporation, Saint Joseph, MI, USA) was to determine the amount of nitrogen (N) components and evaluate the crude protein (CP) content. Van Soest et al [16] outlined a standard method for determining the content of neutral detergent fiber (NDF) and acid detergent fiber (ADF).

### Rumen fluid preparation

Rumen fluid was collected from a sample of four male 4-year-old Thai native beef cattle with an average weight of 200±50 kg. The cattle were housed in separate pens and provided with a daily ration of concentrate that constituted at 0.5% of body weight. Additionally, animal s were given *ad libitum* access to rice straw at 7:00 a.m. and 4:00 p.m. for a period of seven days prior to the collection of rumen fluid. On the final day of the seven-day period, collected 1,000 mL of rumen fluid from each animal before morning feeding. The rumen fluid was filtered through four layers of cheesecloth into pre-warmed thermos flasks, mixed the rumen fluid from each collected animal, and then transported to the laboratory. Table 1 presents a description of the ingredients and chemical composition of the concentrate and rice straw.

### Study design and *in vitro* fermentation

The design of the experiment was a completely randomized design (CRD). Four levels of mMARABAC supplementation at 0, 5, 10, and 15 mg/500 mg on DM basis. The dietary substrate had a 60:40 roughage to concentrate ratio, and the roughage source was rice straw. The rice straw and concentrate were ground to a particle size that could pass through a 1 mm sieve using a blender before being utilized for *in vitro* gas production testing. The gas production experiment was performed with three replicates for each treatment, along with an additional three replicates for the control treatment. In total, 15 serum bottles were created (3 replicates×4 treatments+3 replicates of blank). Measurement of rumen fermentation parameters, specifically NH_3_-N, VFAs, and microbial diversity, was carried out at two sampling time points: 12 hours and 24 hours of incubation. A total of 20 bottles were arranged for this experiment (2 replicates×4 treatments×2 sampling times+2 replicates of blank). An additional twenty bottles were made to investigate the IVDMD at two different sampling times, namely 12 hours and 24 hours of incubation. This was achieved by conducting two replicates for each of the four treatments, as well as including two replicates of a blank sample. A quantity of 500 mg of substrate was weighed and transferred into 50 mL serum bottles. Subsequently, mMARABAC was added in different levels ranging from 0, 5, 10, and 15 mg based on a dry matter basis. Following the addition of substrates, the bottles received a CO_2_ flushing procedure and were subsequently injected with 40 mL of artificial saliva. The artificial saliva was made in accordance with the methodology outlined by Menke and Steingass [17]. Following the injection, the serum bottles were sealed with an aluminum lid and subjected to incubation at a temperature of 39°C until the time of sampling.

### Sampling collection and gas production analysis

A sequence of incubation durations involving 1, 2, 4, 6, 8, 12, 18, 48, 72, and 96 hours was conducted to assess gas production. The gas generated during each incubation period was utilized to investigate the accumulation of gas by using the Ørskov and McDonald [18] equation (1) model.


(1) $$\text{Y}=\text{a}+\text{b }(1-{\text{e}}^{\left(-\text{ct}\right)})$$

In the given context, the variable “a” represents the gas production derived from the fraction that is immediately soluble, measured in milliliters (mL). Similarly, the variable “b” represents the gas production derived from the fraction that is insoluble, also measured inmL. The variable “c” denotes the gas production rate constant for the insoluble fraction, measured in mL per hour (mL/h). The variable “t” represents the duration of incubation time, measured in hours. The sum of “a” and “b” represents the potential extent of gas production, measured in mL. Lastly, the variable “Y” denotes the amount of gas produced at specific time points “t”, measured in mL. The serum bottles were taken out of the incubator after 12 and 24 hours of incubation, and their pH levels were promptly tested using a HANNA pH meter manufactured by HANNA instruments in Romania. Subsequently, the fermentation liquid received filtration using a four-layered cheesecloth, resulting in its division into two separate phases. The liquid phase was utilized for the analysis of NH_3_-N and VFAs concentrations, while the solid part was designated for the examination of the microbial population. The first part of the samples underwent centrifugation for 15 minutes at 16,000×g, and then the supernatant was collected and kept at −20°C until analysis. The concentration of NH_3_-N was determined utilizing the micro-Kjeldahl method as described in the study conducted in AOAC [19]. Additionally, the VFAs concentration, which includes C2, propionic acid (C3), and butyric acid (C4), was measured using gas chromatography (GC) with a GC-2014 (Shimadzu, Kyoto, Japan) according to So [20]. The subsequent set of samples was appropriately managed and maintained in a refrigerated environment in preparation for DNA analysis according to Koike and Kobayashi [21]. After 12 and 24 hours of incubation in the IVDMD study, serum bottles were used to collect gases for the study of CH_4_ production. A volume of 10 mL of gas was obtained by means of a 10 mL syringe. This gas was subsequently introduced into serum bottles with a capacity of 25 mL, which were sealed using a combination of rubber lids and aluminum caps. The bottles were then securely covered with parafilm. The measurement of CH_4_ production was conducted using GC (specifically, the GC-17A System; Shimadzu). The gas chromatograph was equipped with a thermal conductivity detector and a Shin carbon column with dimensions of 3 m×3 mm. The activated charcoal used in the column had a mesh size of 60/80 and was sourced from Kyoto, Japan. The methodology employed for CH_4_ measurement followed the work of Sittijunda et al [22].

Following a 12-hour and 24-hour incubation period, the serum bottles were taken out of the incubator and subsequently subjected to freezing for preservation until further examination. The IVDMD was computed following the methodology outlined in the study conducted by Menke and Steingass [17]. In a concise manner, the serum vials were extracted from the freezer and subsequently thawed at room temperature. Afterwards, the fermentation liquid samples were filtered using Gooch crucibles with a porosity of 40 mm, which were pre-weighed before the filtration process. The filtered Gooch crucibles were then subjected to oven-drying at a temperature of 100°C for a duration of 24 hours. Following the drying process, the Gooch crucibles were weighed, and the residual DM was determined. In order to determine IVDMD, the DM values of the residue were subtracted from the DM of the blank residue.

### Statistics

The data were analyzed using the generalized linear model technique of SAS [23] in a CRD. The data were subjected to analysis using the specified model.


Yij=m+Mi+1ij

The variable Yij represents the observed data, m denotes the average value, Mi represents the impact of the distinct mMARABAC levels (with i ranging from 1 to 4), and 1ij represents the residual effect. The findings are reported in the form of mean values, accompanied by the standard error of the means. The determination of differences between treatment means was conducted using Tukey’s test. Statistically significant differences were accepted when the p-value was less than 0.05 (p<0.05). Orthogonal polynomials were employed to analyze the trends of mMARABAC-level responses.

## RESULTS

### Chemical composition of the experimental diets

Table 1 displays the chemical composition of the concentrate, rice straw, and mMARABAC. The concentrate and rice straw contained 14.6% and 2.4% CP, respectively. The mMARABAC contained 3.02 TPC g GAE/kg DM, 0.16 TFC g QUE/kg DM, 76.6% DPPH radical inhibition, 79.7% ABTS radical inhibition, and 22.34 FRAP g TROE/kg DM).

### Gas production kinetics and *in vitro* degradability of dry matter

The gas production kinetics are shown in Table 2. Supplementation of mMARABAC significantly affected the insoluble fraction of gas production (b), rate of gas production value (c), and potential extent of gas (|a|+b) value (p<0.05), while the soluble fraction of gas production (a) was not influenced by the treatments. Furthermore, the mMARABAC increased the cumulative gas at 96 h after incubation (p<0.05) when the mMARABAC was supplemented with 5 mg of the substrate in comparison to 0 mg of mMARABAC. However, adding mMARABAC to the diet had no effect on the IVDMD (p> 0.05).

### Ruminal pH, ammonia-nitrogen concentration and methane production

The effects of mMARABAC supplementation on pH, NH_3_-N concentration and CH_4_ production are presented in Table 3. mMARABAC supplementation had no effect on ruminal pH and NH_3_-N (p>0.05). Nevertheless, the level of mMARABAC supplementation in the diet, which ranged from 5 to 15 mg of the substrate, resulted in a decrease in CH_4_ production at 24 hours (p<0.05).

### Ruminal volatile fatty acids

The effectiveness of mMARABAC supplementation on total VFAs, C2, C3, C4, C2:C3 ratio are presented in Table 4. The concentration of total VFAs, C2, C3, C4, and the C2:C3 ratio were not influenced by mMARABAC supplementation (p> 0.05).

### Microbial diversity

Table 5 shows the effects of mMARABAC supplementation on microbial diversity. The effect of mMARABAC supplementation on *Fibrobacter succinogenes*, *Ruminococcus albus*, *Ruminococcus flavefaciens*, *Butyrivibrio fibrisolvens* and *Megasphaera elsdenii* differed between treatments (p>0.05). While, the mMARABAC supplement did not demonstrate a statistically significant effect on methanobacterial (p<0.05).

## DISCUSSION

The supplementation with mMARABAC had an impact on the gas production rate constant of the insoluble fraction (c). The study conducted by Sommai et al [24] showed a substantial impact of flavonoid extract (FE) supplementation on the gas production rate constantly associated with the insoluble fraction (c). The quadratic increase in FE level has a significant impact on the (c) value. Ørskov and McDonald [18] established a negative association between the potential extent of gas (a+b) and the gas production rate constant (c) of the insoluble fraction (b). This suggests that an augmentation in the potential extent of gas (a+b) relates to a reduction in the gas production rate constant (c) of the insoluble fraction (b). Oskoueian et al [25] found that when the substrate had 4.5% (w/w) of naringin, rutin, and quercetin, there was a reduced the insoluble fraction of gas production (b) and rate of gas production value (c). Purba et al [26] reported that 15 to 30 mg of piper betle powder, combining 0, 15, and 30 mg of sunflower oil, an abundant source of flavonoids and their aromatic derivatives, increased the total gas production from fermentation. According to Oskoueian et al [25], the effects of flavonoids and phenolic compounds had an effect on gas production. According to the results shown in the experiment, supplementation with mMARABAC did not affect IVDMD, similar to Sommai et al [24], and supplementation with Brazilian spinach pellet affected nutrient digestibility. There were no significant differences among treatments. This data is related to that of Prommachart et al [27], discovered that the anthocyanin extracted from purple corn and black rice contains both phenolic acids and anthocyanin. Supplementation of up to 6% in diets did not affect DM, organic matter, NDF, and ADF digestibility. Therefore, the anthocyanin concentration in treatment diets might not be high enough to interfere with microbes in the rumen. In addition, Hosoda et al [28] demonstrated that anthocyanin corn silage did not affect total digestibility of nutrient intake in lactating dairy cows.

The fact that fermentation in the rumen enhanced in a positive manner suggests that the bioactive compounds and the amounts added are not changing the pH and NH_3_-N levels in the rumen in this experiment. The ruminal pH value ranged from 6.7 to 6.9 and was within the normal value as reported as the optimal pH ranges from 6.5 to 7.0 [29]. However, it was found that it could maintain the pH balance in the rumen. Wanapat and Pimpa [30] stated that NH_3_-N concentrations ranging between 15 to 30 mg/dL were suitable for ruminal microbial activity, improving rumen ecology. In this experiment, the concentration of NH_3_-N did not change between treatments because the concentrate diet contained the same amount of protein. The previous experiment found a similar correlation between a higher crude protein intake and a higher NH_3_-N concentration. Phenolic compounds can bind and precipitate macromolecules, such as dietary proteins, thereby reducing protein digestibility [31]. The phenolic structure of green tea could explain why green tea supplementation does not reduce NH_3_-N concentration. The catechins are low-molecular-weight phenols. In contrast to low-molecular-weight phenols, which can’t precipitate proteins, phenolic compounds can bind to proteins because their phenolic polymers have been highly hydroxylated. Sarni-Manchado et al [32] found that polymeric condensed tannins bound proteins more effectively than low-molecular-weight oligomers and monomers. Nevertheless, Aemiro et al [33] found that the levels of NH_3_-N in vitro decreased as the level of green tea extract increased.

This experiment demonstrated that the mMARABAC supplementation had an impact on CH_4_ production. It is suggested that it is the impact of phytonutrient feeding which reduces CH_4_. Throughout the fermentation process, the production of VFAs, CO_2_, and metabolic hydrogen (H_2_) proceeds. In this process, CO_2_ serves as the carbon source, while H_2_ acts as the primary electron donor. The chemical equation for methane production is CO_2_+4H_2_ → CH_4_+2H_2_O. Methanogenic archaea then utilize these substances to synthesize CH_4_ [34]. Moreover, it has been demonstrated by Wanapat et al [34] that certain condensed tannins possess the ability to mitigate CH_4_ production. According to Patra and Saxena [35] reported that both condensed tannins and hydrolysable tannins have anti-methanogenic properties. Tannins have two different modes of action: a direct impact on methanogens in the rumen and an indirect impact on H generation due to less feed degradation. Although their impact on methanogens doesn’t necessarily correspond with their impact on protozoa, saponins lower the population of some methanogens linked to protozoa [36]. The antiprotozoal effect of saponins is correlated with the interaction between the sterol moiety and saponin found in the protozoa membrane [35]. Hence, Chaya leaf pellet supplementation is highly promising for ruminant feeding [37]. According to Viennasay et al [38], the ratio of roughage: concentrate at 30:70 with bamboo grass pellet supplementation could reduce CH_4_ production, while interactive effects were additionally observed. The administration of the supplement demonstrated a significant (p<0.01) impact on reducing CH_4_ generation in the rumen.

In the results shown in the experiment, supplementation with mMARABAC did not affect VFAs. Furthermore, Cherdthong et al [39] indicated that the supplementation of *Piper sarmentosum* leaf powder had no effect on C2, C4, or the C2:C3 ratio. In addition, also other authors Calabrò et al [40] reported the same VFA production even with significantly different cumulative gas production. Ampapon et al [41] discovered that total VFAs, C2, and C4 were the same among treatments when using phytonutrient pellets in beef cattle. Several factors have the potential to influence the generation of VFAs, including the composition of the substrate, the availability of the substrate, and the population of microorganisms.

Under this investigation, the microbial diversity of mMARABAC supplementation on *F. succinogenes, R. albus, R. flavefaciens, B. fibrisolvens* and *M. elsdenii* was significantly different by mMARABAC supplementation. Several studies have shown the beneficial effects of bioactive compounds (saponins, tannins, essential oils, and compounds containing organosulfur) on animal production and health, as well as rumen fermentation, including the microbial population [42]. Patra and Saxena [43] demonstrated an increase in the diversity of *F. succinogenes* under the influence of all flavonoids, whereas the populations of *R. albus* and *R. flavefaciens* declined under the same conditions as compared to the control group. In comparison to the control group, higher rumen pH values were observed in high-grain heifers treated with flavonoids. This is probably because bioactive compounds have a positive influence on *M. elsdenii* and other lactate-consuming bacteria [44]. Seradj et al [45] found that a commercial citrus extract with a flavonoid blend (Bioflavex) increased the amount of propionate and the number of *M. elsdenii* in a test tube while decreasing CH_4_ production and the number of hydrogenotrophic methanogenic archaea. Additionally, Morgavi et al [46] demonstrated that there is no expected symbiotic relationship between rumen bacteria and methanogens. However, methanogens can integrate into bacterial biofilms on feed particles, which represents a form of interaction. Methanogens use the CO_2_ and H_2_ produced by most fermentative ruminal bacteria as substrates. As a result, rumen bacteria and methanogens have a mutualistic interaction via the transfer of H_2_ between species. Co-cultures of methanogens with *R. albus, R. flavefaciens*, and *Selenomonas ruminantium* have demonstrated this interspecies H_2_ transfer. The rumen microbiome’s interaction with methanogens affects energy conservation, VFA profiles, and CH_4_ production. [47]. However, Para et al [48] reported that extracts containing phenolics were shown to reduce the emission of CH_4_ in the rumen as well as the count of protozoa. However, it was noted that these extracts did not demonstrate significant efficacy in inhibiting ruminal methanogenesis. This study also found that the methanobacterial population was not different from mMARABAC supplementation.

## CONCLUSION

This experiment concluded that mMARABAC supplementation at 5 mg/500mgDM substrate affected gas production, cumulative gas, *F. succinogenes*, *R. albus*, *R. flavefaciens*, *B. fibrisolvens*, *M. elsdenii and* reduced CH_4_ production. Therefore, ruminants could use mMARABAC supplementation as an agricultural by-product and a source of phytonutrients. However, an *in vivo* study is required to validate the current findings.

## Figures and Tables

**Table 1:** Ingredients and chemical composition of substrate used in this study

Item	Concentrate	Rice straw	mMARABAC
Ingredients (% as fed)			
Cassava chip	54		
Rice bran meal	17		
Palm kernel meal	13		
Soybean meal	10.5		
Urea	2.5		
Sulfur	1		
Salt	1		
Mineral mixed^1)^	1		
Chemical composition			
Dry matter (%)	90.5	89.4	91.2
Organic matter (%DM)	92.2	85.4	98.1
Crude protein (%DM)	14.6	2.4	16.9
Neutral detergent fiber (%DM)	20.5	78.9	54.3
Acid detergent fiber (%DM)	8.2	52.6	13.3
Phytonutrient compound			
TPC (g GAE/kg DM)			3.0
TFC (g QUE/kg DM)			0.2
DPPH radical inhibition (%)			76.6
ABTS radical inhibition (%)			79.7
FRAP (g TROE/kg DM)			22.3

1) Contains per kilogram premix = 10,000,000 IU vitamin A; 70,000 IU vitamin E; 1,600,000 IU vitamin D; 50 g Fe; 40 g Zn; 40 g Mn; 0.1 g Co; 10 g Cu; 0.1 g Se; 0.5 g I.

mMARABAC, microencapsulated phytonutrients; TPC, the total polyphenolic content; TFC, total flavonoid content; DPPH, the 2, 2-diphenyl-1-picrylhydrazyl radical scavenging method; ABTS, the 2, 2′-azino-bis (3-ethylbenzthiazoline-6-sulphonic acid); FRAP, the ferric reducing antioxidant power; TROE, trolox equivalents.

**Table 2:** Effect of mMARABAC supplementation on gas kinetics, cumulative gas production (96 h) and *in vitro* true dry matter degradability (IVDMD)

mMARABAC mg/500 mg DM	Gas production kinetics^1)^				Cumulative gas at 96 h (mL/500 mg DM)	IVDMD (%)	

	a	b	c	|a|+b		12 h	24 h
0	−1.0	60.2^ab^	0.03^b^	61.2^ab^	63.6^a^	65.1	80.8
5	−0.6	70.3^a^	0.03^b^	70.9^a^	76.4^a^	63.4	79.4
10	−0.9	48.3^b^	0.05^a^	49.2^b^	52.6^b^	68.2	82.9
15	−1.5	42.5^b^	0.05^a^	44.0^b^	47.1^b^	68.1	82.8
SEM	0.20	3.61	0.002	3.65	3.96	1.09	1.46
Orthogonal polynomials							
Linear	0.29	0.03	0.002	0.03	0.04	0.12	0.36
Quadratic	0.21	0.24	0.31	0.27	0.22	0.64	0.76
Cubic	0.83	0.12	0.11	0.13	0.11	0.17	0.39

1) a, gas production from immediately soluble fraction; b, gas production from insoluble fraction; c, gas production rate constant for insoluble fraction; |a|+b, potential extent of gas.

a,b Means within a column with different superscript letter are significantly different (p<0.05).

mMARABAC, microencapsulated phytonutrients; DM, dry matter; SEM, standard error of mean.

**Table 3:** Effect of mMARABAC supplementation on ruminal pH, ammonia-nitrogen and methane production

mMARABAC mg/500 mg DM	pH			NH_3_-N (mg/dL)			CH_4_ (mL/100 mL of total gas)		

	12 h	24 h	Mean	12 h	24 h	Mean	12 h	24 h	Mean
0	6.9	6.9	6.9	19.5	19.1	19.3	3.7	3.5^a^	2.2
5	6.8	6.9	6.9	18.9	19.4	19.2	2.7	2.0^b^	2.4
10	6.8	7.0	6.9	17.8	19.8	18.8	4.4	0.9^b^	3.9
15	6.8	7.0	6.9	19.6	19.5	19.6	1.2	0.7^b^	1.2
SEM	0.11	0.02	0.01	0.43	0.70	0.33	1.00	0.41	0.54
Orthogonal polynomials									
Linear	0.13	0.68	0.18	0.83	0.69	0.77	0.40	0.03	0.61
Quadratic	0.88	0.54	0.72	0.13	0.74	0.44	0.47	0.48	0.11
Cubic	0.35	0.28	0.26	0.28	0.88	0.56	0.31	0.17	0.16

a,b Means within a column with different superscript letter are significantly different (p<0.05).

mMARABAC, microencapsulated phytonutrients; DM, dry matter; NH_3_-N, ammonia nitrogen; CH_4_, methane production; SEM, standard error of mean.

**Table 4:** Effect of mMARABAC supplementation on volatile fatty acids

mMARABAC mg/500 mg DM	C2:C3 ratio			VFAs (mol/100 mol)									Total VFAs (mmol/L)		

				C2			C3			C4					
				
	12h	24h	Mean	12h	24h	Mean	12h	24h	Mean	12h	24h	Mean	12h	24h	Mean
0	1.6	0.8	1.1	51.6	40.1	45.9	32.1	49.6	40.9	16.3	10.3	13.3^a^	35.3	63.5	44.4
5	2.2	0.8	1.3	59.8	40.9	50.4	28.2	53.9	39.1	13.0	9.2	9.3^c^	35.2	66.9	55.7
10	1.8	0.8	1.2	54.8	40.2	47.5	31.4	49.4	40.4	13.8	10.4	12.1^ab^	34.3	50.0	44.6
15	2.1	0.8	1.3	59.2	40.2	49.7	28.4	50.7	39.6	12.4	9.1	10.8^b^	35.6	55.7	45.7
SEM	0.16	0.02	0.05	2.26	0.67	1.12	1.32	1.78	0.73	0.79	0.43	0.20	1.18	3.49	2.66
Orthogonal polynomials															
Linear	0.39	0.67	0.42	0.28	0.95	0.28	0.39	0.93	0.61	0.10	0.38	0.03	0.98	0.14	0.69
Quadratic	0.70	0.66	0.59	0.58	0.72	0.51	0.81	0.58	0. 67	0.45	0.87	0.02	0.68	0.82	0.24
Cubic	0.17	0.91	0.21	0.19	0.67	0.15	0.18	0.26	0.31	0.28	0.15	0.01	0.70	0.12	0.11

a–c Means within a column with different superscript letter are significantly different (p<0.05).

mMARABAC, microencapsulated phytonutrients; DM, dry matter; C2:C3, acetate to propionate ratio; VFAs, volatile fatty acids; C2, acetate; C3, propionate; C4, butyrate; SEM, standard error of mean.

**Table 5:** Effect of mMARABAC supplementation on microbial diversity

mMARABAC mg/500 mg DM	*Fibrobacter succinogenes* (Log copies/mL)			*Ruminococcus albus* (Log copies/mL)			*Ruminococcus flavefaciens* (Log copies/mL)			*Megasphaera elsdenii* (Log copies/mL)			*Butyrivibrio fibrisolvens* (Log copies/mL)			*Methanobacterial* (Log copies/mL)		
					
	12 h	24 h	Mean	12 h	24 h	Mean	12 h	24 h	Mean	12 h	24 h	Mean	12 h	24 h	Mean	12 h	24 h	Mean
0	7.7^b^	8.6^a^	8.1	8.9	9.8^a^	9.3	8.3^ab^	9.0^a^	8.6^a^	8.6^b^	9.2^a^	8.9	8.7^ab^	9.0	8.9^ab^	9.5	9.8	9.6
5	8.8^a^	7.9^c^	8.3	9.5	9.4^ab^	9.3	8.8^ab^	8.2^b^	8.5^ab^	8.8^ab^	8.6^ab^	8.7	8.9^ab^	9.1	9.0^ab^	9.5	9.7	9.6
10	8.6^ab^	8.5^ab^	8.5	9.3	9.2^b^	9.3	9.1^a^	7.3^c^	8.2^ab^	9.7^a^	8.1^b^	8.9	9.4^a^	8.8	9.1^a^	9.9	9.6	9.7
15	8.6^ab^	7.9^bc^	8.3	9.1	9.1^b^	9.1	7.8^b^	8.0^bc^	7.9^b^	8.2^b^	8.3^b^	8.2	8.4^b^	9.0	8.7^b^	9.5	9.6	9.6
SEM	0.18	0.10	0.05	0.12	0.07	0.11	0.23	0.14	0.11	0.17	0.11	0.11	0.13	0.04	0.09	0.09	0.06	0.06
Orthogonal polynomials																		
Linear	0.09	0.12	0.12	0.72	0.01	0.28	0.48	0.01	0.02	0.93	0.01	0.66	0.61	0.48	0.46	0.40	0.15	0.84
Quadratic	0.09	0.53	0.06	0.06	0.23	0.60	0.04	0.02	0.67	0.03	0.06	0.22	0.03	0.65	0.05	0.11	0.44	0.34
Cubic	0.25	0.02	0.26	0.43	0.93	0.86	0.39	0.14	087	0.05	0.46	0.13	0.07	0.08	0.31	0.11	0.32	0.39

a–c Means within a column with different superscript letter are significantly different (p<0.05).

mMARABAC, microencapsulated phytonutrients; DM, dry matter; SEM, standard error of mean.
